# UcTCRdb: An unconventional T cell receptor sequence database with online analysis functions

**DOI:** 10.3389/fimmu.2023.1158295

**Published:** 2023-03-13

**Authors:** Yunsheng Dou, Shiwen Shan, Jian Zhang

**Affiliations:** Academy of Medical Engineering and Translational Medicine, Tianjin University, Tianjin, China

**Keywords:** unconventional T cell, MAIT cell, NKT cell, CD8αα T cell, γδ T cell, TCR (T cell receptor), bioinformatics and computational biology

## Abstract

Unlike conventional major histocompatibility complex (MHC) class I and II molecules reactive T cells, the unconventional T cell subpopulations recognize various non-polymorphic antigen-presenting molecules and are typically characterized by simplified patterns of T cell receptors (TCRs), rapid effector responses and ‘public’ antigen specificities. Dissecting the recognition patterns of the non-MHC antigens by unconventional TCRs can help us further our understanding of the unconventional T cell immunity. The small size and irregularities of the released unconventional TCR sequences are far from high-quality to support systemic analysis of unconventional TCR repertoire. Here we present UcTCRdb, a database that contains 669,900 unconventional TCRs collected from 34 corresponding studies in humans, mice, and cattle. In UcTCRdb, users can interactively browse TCR features of different unconventional T cell subsets in different species, search and download sequences under different conditions. Additionally, basic and advanced online TCR analysis tools have been integrated into the database, which will facilitate the study of unconventional TCR patterns for users with different backgrounds. UcTCRdb is freely available at http://uctcrdb.cn/.

## Introduction

T cells play critical roles in immune responses. The classical T cell immune responses are primarily driven by the specific recognition between T cell receptors (TCRs) and peptide antigens presented by major histocompatibility complex (MHC) class I and II molecules. While there are several subpopulations of T cells, which work independently of this TCR-peptide-MHC paradigm, are defined as unconventional T cells. The unconventional T cells interact with molecules presented by MHC class Ib or MHC-like molecules, including lipid moieties, bacteria-derived vitamin B metabolites phosphoantigens, and butyrophilins (1, 2). These innate-like T cell subpopulations, including the MR1-restricted mucosal-associated invariant T cells (MAIT) (3, 4), CD1d-restricted type I natural killer T cells (iNKT) (5, 6), CD1a- (7), CD1b- (8), CD1c- (9), H2-M3- (10), Qa-1- (11) or HLA-E (12)-restricted T cells, TCRαβ CD8αα intraepithelial T lymphocytes (IELs) (13) and TCRγδ T cells (14), typically exhibit relatively simplified TCR patterns, rapid effector responses, and ‘public’ antigen specificities.

Recent advances have highlighted that the recognition of antigens by unconventional TCRs is important for their early-life development and activation (15–19). However, the fine-grained patterns of antigen recognition by unconventional TCRs remain largely unknown. The most widely studied unconventional T cells, such as MAIT and iNKT cells, have been shown to have invariant TCRα gene usage and limited TCRβ gene diversity (3–5, 20, 21). These gene usage biases are most likely due to the nonpolymorphic ligands they recognized, but the role of the randomly generated CDR3 sequences in the interaction is still not well clarified. Moreover, there are some unconventional T cell subpopulations such as TCRαβ CD8αα IELs and TCRγδ T cells, that were characterized by diverse TCR gene usage and CDR3 sequence, but with unknown antigenic specificity (22, 23). With the development of TCR sequencing technology and its wide use in the T cell studies (6, 24–29), further characterizing the unconventional TCR patterns is a critical requirement for understanding their responses to certain antigens and is a fundamental aspect of studying the development of such unconventional T cell subsets. While the small size and lack of standardization of the released unconventional TCR sequences strongly suggest that a comprehensive database aggregating the published data is in urgent need. To the best of our knowledge, there is still no systematic database for users to access and analyze unconventional TCR sequences.

To fill these gaps, we present UcTCRdb, a comprehensive database integrating published unconventional TCR sequences. Users can easily browse and download the data and search for sequences of their interest. To support mining the sequencing data, we also provide a series of TCR conservation analyses, including V and J gene usage, CDR3 length distribution, amino acid usage, sequence logo char, and similarity network displays. In addition, we offer users an option to analyze the datasets of interest individually, by simply entering data or uploading data files. This code-free analysis will facilitate the discovery of sequence patterns for users with different backgrounds. Since existing TCR databases mainly focus on conventional T cells (30–34), we believe that UcTCRdb will be a valuable resource to study unconventional T cells and provide new insights into T cell immunity.

## Materials and methods

### Data collection and preprocessing

To construct a comprehensive database of unconventional TCRs, we searched the published literature for unconventional TCR data on PubMed following the search terms: (CD8αα intraepithelial lymphocytes[Title/Abstract]) OR (mucosal-associated invariant T cells[Title/Abstract]) OR (MR1-restricted T cells[Title/Abstract]) OR (type I NKT T cells[Title/Abstract]) OR (MAIT[Title/Abstract]) OR (γδ T[Title/Abstract]) OR (iNKT[Title/Abstract]) OR (CD1b[Title/Abstract]) AND (T cell receptor). All results were then checked for inclusion of TCR sequences. The corresponding data listed in the literature tables or supplementary files were manually downloaded and archived. For those that do not provide processed TCR data, we downloaded the raw FASTQ files from Sequence Read Archive (SRA) (35) and extracted the TCR sequences using MiXCR (36). Besides, TCR sequences of γδ T cells (dataset ID: 20-36) were collected from the immuneAccess website by setting the locus to TRAD OR TRG in the data browser page (https://clients.adaptivebiotech.com/immuneaccess/browse). To ensure the reliability and quality of the data, all the TCR sequences were subject to quality control by a uniform workflow, and only sequences with high confidence were retained. The sequences which lack V/J gene annotation or contain any illegal amino acid abbreviated characters were discarded. For the sequences that contain multiple V/J gene annotations, the first ones were kept as the most confident annotated gene. Duplicated sequences of identical V gene, J gene, and CDR3 amino acid sequence in each dataset were removed. By re-organizing the data format, the corresponding T cell type information and PubMed ID were retained in addition to the V gene, J gene, and TCR CDR3 amino acid sequences.

### TCR analysis

UcTCRdb allows users to analyze selected data in the database or uploaded data files. V and J gene segment usage and gene-gene pairing landscapes are analyzed and shown using vertical stacks connected by curved paths whose thickness is proportional to the number of TCR clones with the respective gene pairing. The length distribution of the TCR CDR3 amino acid sequences was shown by the normalized frequency of each TCR chain. The normalized mutual information values (NMI) between each pair of CDR3α/β residues were calculated for different CDR3 lengths to represent the association of amino acid usage at different positions. We use Shannon entropy to measure the uncertainty of amino acid usage at different CDR3 positions. The entropy H(i) was calculated according to formula (1), where P(xi) represents the probability amino acid x appearing at position i, and |Xi| is the set of all different amino acids in position i. Then the mutual information (MI) of amino acid distribution for position i and j was calculated according to formula (2), and P(xi, yj) is the joint probability of x in position i and y in position j. The NMI was then calculated by using formula (3).


(1) $$\text{H}\left(i\right)=-\sum_{xi}^{\left|Xi\right|}\text{P}\left(xi\right)\log\left(\text{P}\left(xi\right)\right)$$

(2) $$\text{MI}\left(i,\;j\right)=\sum_{xi}^{\left|Xi\right|}\sum_{yj}^{\left|Yj\right|}\text{P}\left(xi,yj\right)\log\left(\frac{\text{P}\left(xi,yj\right)}{\text{P}\left(xi\right)P\left(yj\right)}\right)$$

(3) NMI(i,j)=2MI(i,j)/(H(i)+H(j))

The NMI takes values in the range of 0-1, with a larger NMI indicating a higher amino acid correlation. We plotted the NMI matrix with a heatmap. The CDR3 sequence logos including amino acids as well as V and J genes were depicted by calculating the occurrence weights of different amino acids at each position. The larger the amino acid char in the sequence logo represents the higher frequency of amino acid occurrence. The amino acids were colored by their chemical properties in the sequence log plot. The scatter plot of amino acid usage was generated by calculating the proportion of each amino acid. To compare the differences between cell subsets, UcTCRdb has included 8 different datasets as references in the analysis module. To visualize the global similarity between TCR sequences, we calculated the Hamming distance between each pair of TCRs. We plotted a network graph where each node represents a TCR sequence and each edge represents a distance of less than 2 between two sequences. The visualization allows users to easily identify and explore groups of highly similar sequences.

### Implementation of UcTCRdb

UcTCRdb was implemented using Vue (version 3.2.13), an approachable, performant, and versatile framework for building web user interfaces and Flask (version 1.1.2), a lightweight Web Server Gateway Interface (WSGI) web application framework. All statistics and figures were calculated using Python (version 3.6.9) scripts and plotted using Echarts (version 5.3.2). The website was proxied on Nginx, an HTTP and reverse proxy server. And the server was running on the AliCloud server.

## Results

### Overview of UcTCRdb

**Figure 1:**
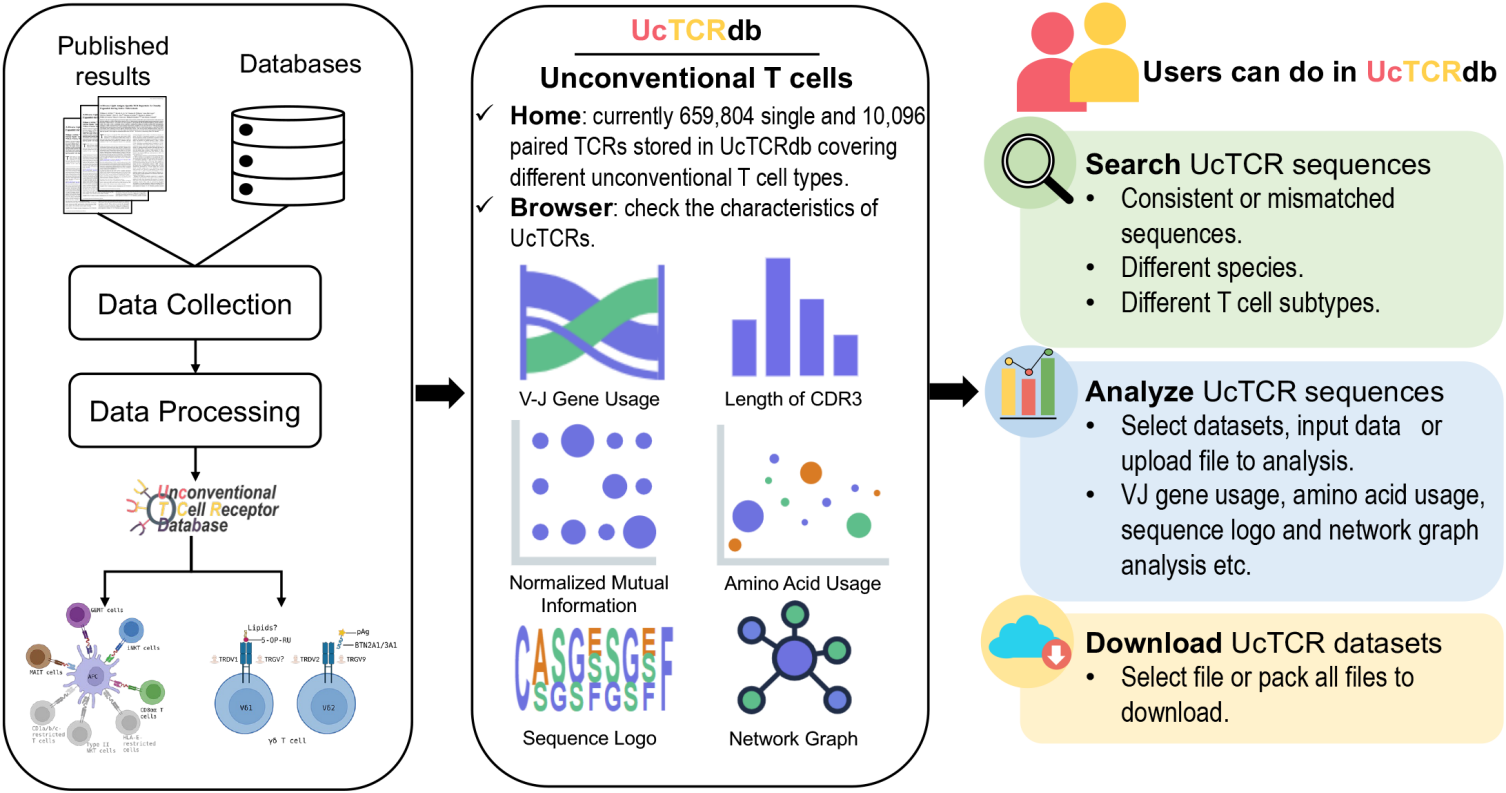
Overview of UcTCRdb workflow and modules. All the data were obtained from the publicly available database and published literature. A standard data processing pipeline was used to clean and filter the data. A total of 669,900 unconventional TCRs passed the processing criteria and were stored in the database. Their basic information is displayed in the Home and Browser modules. The other three main modules in the database provide data analysis, retrieval, and download functions respectively.

UcTCRdb consists of five main modules: Home, Browser, Analysis, Search, and Download (**Figure 1**). Users can access the introduction, significant functions, and summarization of the database from the home page. The browser module allows users to interactively look through the characteristics of TCRs in different unconventional T cell subpopulations from different species. To facilitate the visualization and comparison of the TCR characteristics between different datasets, UcTCRdb provides a codeless analysis module. Users can easily analyze the repertoire data without any coding experience by selecting the datasets in UcTCRdb or uploading their data files, which will provide a great convenience for users with no programming background to quickly analyze and grasp the features of TCRs. As a comprehensive database, one of the core applications of UcTCRdb is to allow users to search for identical or similar TCR sequences by specifying species and cell types. In addition, the download page offers a user-defined manner that allows users to directly select and download the data.

Based on the data processing criteria, a total of 659,804 single-chain TCR sequences and 10,096 paired-chain TCR sequences from 34 corresponding studies in humans, mice, and cattle were collected and integrated into UcTCRdb (**Supplemental Table 1**). Among them, over 85% of the data were derived from human samples (**Supplementary Figure 1A**) and are mostly γδ TCRs (**Supplementary Figures 1B, C**). This was expectable since we retrieved most datasets from studies on the human γδ T cells. Besides, MR1-restricted MAIT cells, CD1d-restricted iNKT cells, and CD1b-restricted germline-encoded mycolyllipid-reactive (GEM) T cells, which have invariant TCRα gene usage and limited TCRβ diversity, account for the majority of unconventional TCRαβ datasets (**Supplementary Figure 1D**). Moreover, two datasets of TCRαβ^+^CD8αα^+^ IELs were also included in the database.

### Interactively browse the features of unconventional TCRs

**Figure 2:**
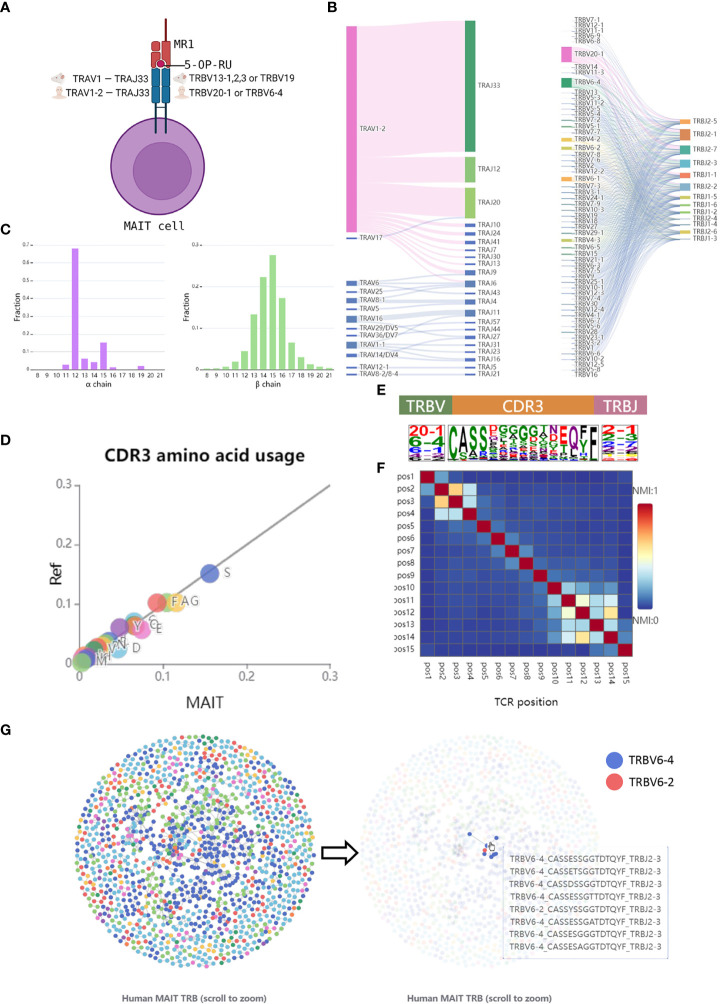
Browse the features of MAIT TCRs. **(A)** A schematic representation of MAIT cell. **(B)** V and J gene segment usage and gene-gene pairing landscapes are shown using vertical stacks (one for each V and J segment) connected by curved paths whose thickness is proportional to the number of TCR clones with the respective gene pairing. **(C)** CDR3 amino acid length distribution per chain. **(D)** The CDR3β sequence logos including amino acids as well as TRBV and TRBJ were depicted by calculating the occurrence weights of different amino acids at the same position. The amino acids were colored by their chemical properties. **(E)** Amino acid usage between MAIT and conventional TCRβ CDR3 sequences. A total of 100,000 conventional TCRβ sequences were randomly selected from the dataset (37). **(F)** Heatmap showing the normalized mutual information value (NMI) between each pair of CDR3β residues. The NMI takes values in the range of 0-1, with a larger NMI indicating a higher amino acid correlation. **(G)** The network graph is obtained by calculating the Hamming Distances between different CDR3 sequences. Nodes, which represent TCR clones, were colored by different TRBV genes. Edge was drawn if the distance between two clones is less than 2.

The browser page focuses on the features of TCRs in the database, which allows users to look through the analysis results of the data from different views. To optimize the demonstration of the sequences data, unconventional T cells were categorized into two groups, unconventional αβ T cells, and γδ T cells. Here, we take human MAIT TCRs as an example to introduce the browser module (**Figure 2A**). Consistent with the previous studies (26, 38), we observed invariant TCRα gene usage and limited TCRβ diversity in the integrated MAIT TCR data. As expected, the dominant conserved TCRα gene is TRAV1-2 and TRAJ33 (**Figure 2B**) and the length distribution of TCRα CDR3 is concentrated at 12 amino acids (**Figure 2C**). In contrast, MAIT TCRβ shows more diversified gene usage and CDR3 length (**Figures 2B, C**). As shown in **Figure 2B**, the dominant TCRβ V genes are TRBV20-1, TRBV6-1,2,4, and TRBV4-1,2 suggesting that the usage bias of these genes might be related to antigen recognition by MAIT cells.

To further display the conservation of MAIT TCRβ sequences, we select the CDR3 sequences with a length of 15 amino acids, which represents the highest length frequency, for downstream analysis. By calculating the amino acid fraction and comparing it to the controlled conventional TCRβ CDR3s, we found differences in the distribution of amino acid usage between them, particularly the higher use frequency of glycine (G) and Glutamic acid (E) in MAIT TCRβ CDR3 sequences (**Figure 2D**). The heatmap of CDR3 NMI scores falls into three blocks, corresponding to the V gene motif region, the CDR3 highly variable region, and the J gene motif region (**Figures 2E, F**). In addition, multiple clusters were found in the similarity network graph of MAIT TCRβ sequences, which also indicated the conserved patterns of MAIT TCRβ (**Figure 2G**). The above examples showed that users can easily access TCR sequence features of different unconventional T cells across different species on the UcTCRdb browser page.

### Analysis module in UcTCRdb

**Figure 3:**
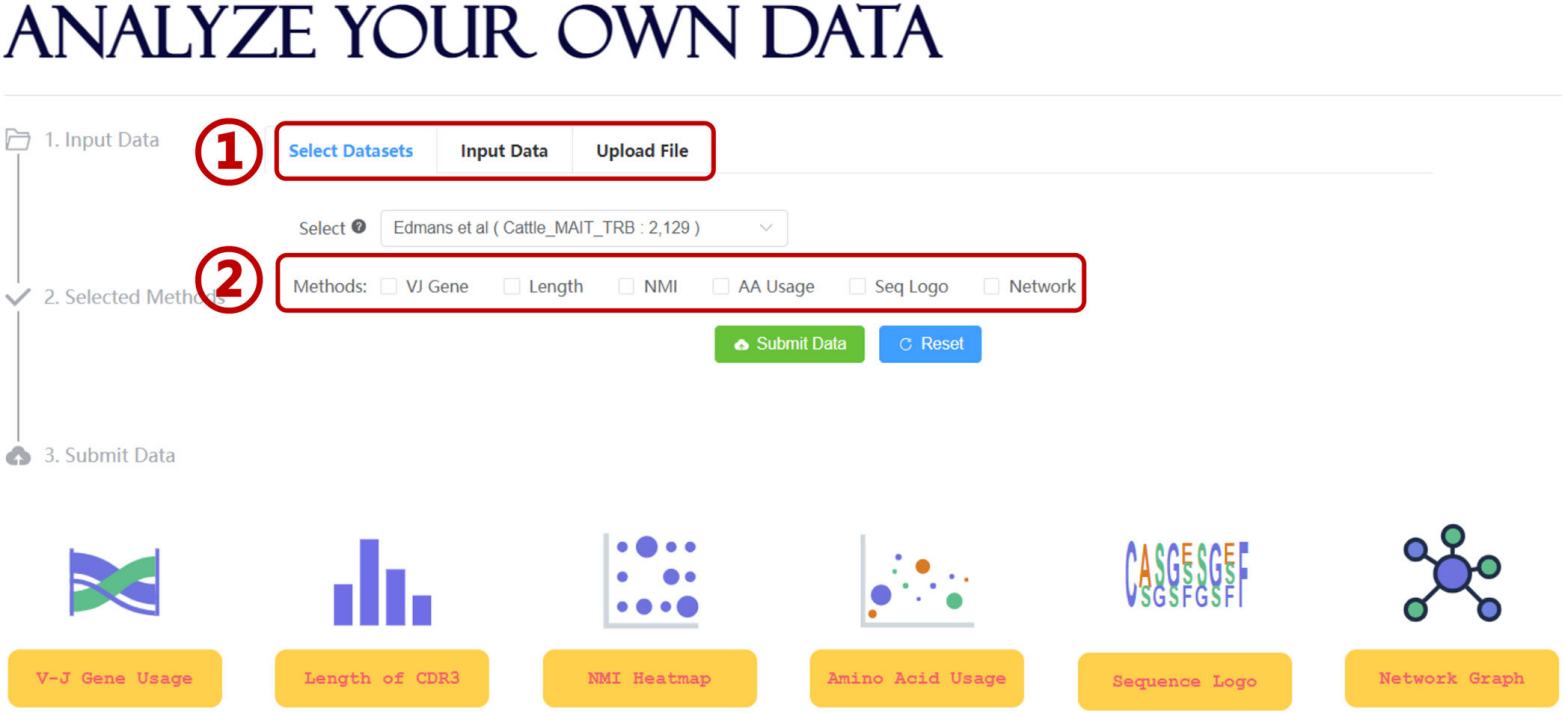
Analysis module in UcTCRdb. Screenshot of the search analysis page (1). UcTCRdb allows users to select a specific dataset or upload their data for analysis (2). There are six functions including TCR gene usage, CDR3 length distribution, NMI, amino acid usage, sequence conservation, and similarity network calculation in this module. Users can choose any one or several of them for data analysis.

Further data mining of the unconventional TCR repertoire is critical for improving our understanding of the interaction between UcTCRs and their corresponding antigens. To simplify the analysis workflow and facilitate more users, UcTCRdb integrated several TCR analysis tools (**Figure 3**). For an overall analysis of the UcTCRs, the length distribution of CDR3 and Sankey graph display of VJ gene usage were provided to access the intrinsic characters of the data. In addition, the NMI heatmap and sequence logo char of the CDR3 sequences were provided to depict the correlation and probability of amino acids in each position. The NMI takes values from 0-1, with a larger number indicating a higher amino acid correlation.

To identify the differences between UcTCRs and conventional TCRs, we compared the amino acid usage between unconventional TCRs and background TCRs [(100,000 sequences randomly sampled from the conventional TCR CDR3 sequences (37)]. The result can be visualized by a scatter diagram with the horizontal and vertical coordinates indicating the amino acid proportion in UcTCRs and references. To obtain the global similarity of TCR s, we calculated the Hamming distance between any pair of CDR3 sequences and constructed a distance matrix. The results were visualized *via* a network graph, where nodes represent TCR sequences, and edges were drawn if the distance between two nodes is less than 2. Nodes were further colored by different TRBV genes. Users can click on the node to trigger all adjacent nodes and get the corresponding sequence information. The above tools were all integrated into the analysis interface of UcTCRdb, allowing for personalized analysis on both selected datasets and uploaded data files (**Figure 3**).

### Search and download sequence data

**Figure 4:**
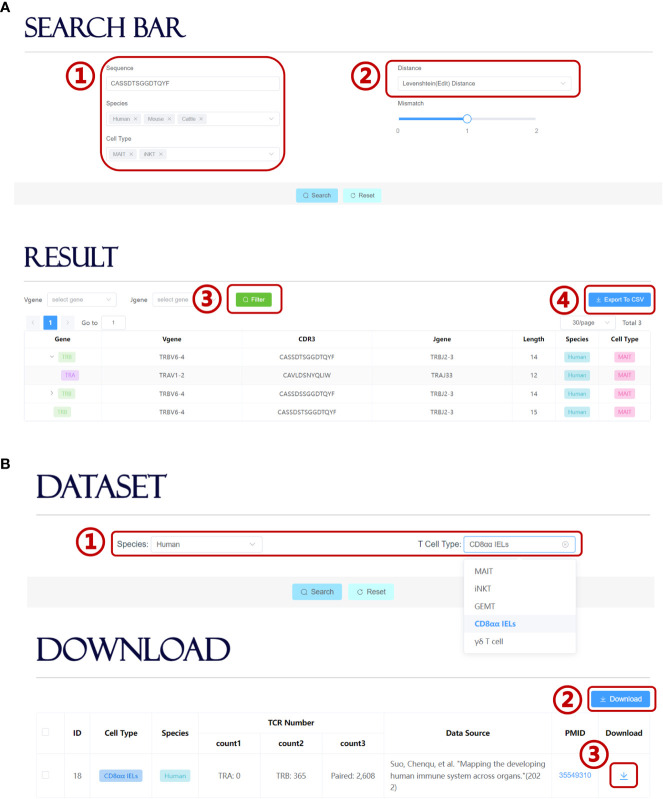
Search module and download module in UcTCRdb. **(A)** Screenshot of the search module page (1). Input the query sequence and set species and cell type conditions (2). Select a preset distance calculation method for fuzzy search (3). UcTCRdb allows a secondary filter performed to the result with a specific V or J gene usage by clicking on the filter button (4). The search results can be downloaded by clicking on the ‘Export To CSV’ button. **(B)** Screenshot of the download module page (1). Select unconventional TCR datasets in different species and cell subpopulations (2). Users can download all the selected datasets by directly clicking the download button or down individual datasets one by one (3).

UcTCRdb allows users to search for TCR sequences in the database that is identical or highly similar to the query sequence. Users can set different query conditions, such as specific cell types, species, or whether contain user-defined mismatches (**Figure 4A**). Currently, UcTCRdb provides two options for calculating sequence similarity, i.e. Hamming distance and Levenshtein (edit) distance. The search result can be further filtered by a specific V or J gene. Due to different sequencing methods, the unconventional TCR sequence data that we obtained contain both single-chain and paired chains. We use an asynchronous loading technique to fetch the corresponding pairing chain data so that the results can be reformatted before being displayed. Users can click the display button to view the result and can download the search result by directly clicking the ‘Export To CSV’ button.

On the download page, UcTCRdb enables users to download datasets that are specified by species and cell types (**Figure 4B**). For the convenience of the users who are interested in processing the unconventional TCR sequence data, UcTCRdb also allows users to download all selected datasets by clicking the ‘Download’ button.

## Discussion

Sequencing technology has been widely used in T cell studies, which provide a deep view of the TCR repertoire in many aspects and a massive data source for integrated analysis (24, 37, 39–42). In this study, we systemically collected more than 0.6 million unconventional TCRs from corresponding studies in humans, mice, and cattle and constructed a database with online analysis tools. This database fills the gap that lakes a comprehensive aggregation of unconventional TCR sequence data. Users can easily browse and download the data and search for sequences of their interest. Given that more unconventional T cell subpopulations will be isolated and sequenced (24), we will continue collecting valid unconventional TCR sequence data and updating UcTCRdb. With the extensive unconventional TCR data and user-friendly web tools, UcTCRdb will be a powerful resource for users studying unconventional T cell immunity.

TCR signaling is the key for conventional T cells to survive positive and negative selection in the thymus, which largely depends on the binding affinity of TCR to self-peptide-MHCs. However, how unconventional T cells undergo selection and differentiation during T cell development remains unclear (43). Recent studies on MAIT cells suggested that effective antigen simulation of these cells is essential for their thymic development and activation (44, 45), indicating that the TCRs may also determine the fate of conventional and unconventional T cells. Our study provides a systematic database of current available unconventional TCRs, which might be a valuable resource for understanding the development of unconventional T cells and how they may be related to diseases.

## Data availability statement

The original contributions presented in the study are included in the article/**Supplementary Material**. Further inquiries can be directed to the corresponding author.

## Author contributions

YD and SS collected the data. YD built the database and online analysis platform. JZ supervised the study and wrote the manuscript together with YD. All authors contributed to the article and approved the submitted version.
