# Supplementary material for: UcTCRdb: An unconventional T cell receptor sequence database with online analysis functions
Source: Front Immunol. 2023 Mar 13;14:1158295. doi: 10.3389/fimmu.2023.1158295 (PMC10040587; doi:10.3389/fimmu.2023.1158295)
Supplement: Supplementary file 2 [file DataSheet_1.pdf]

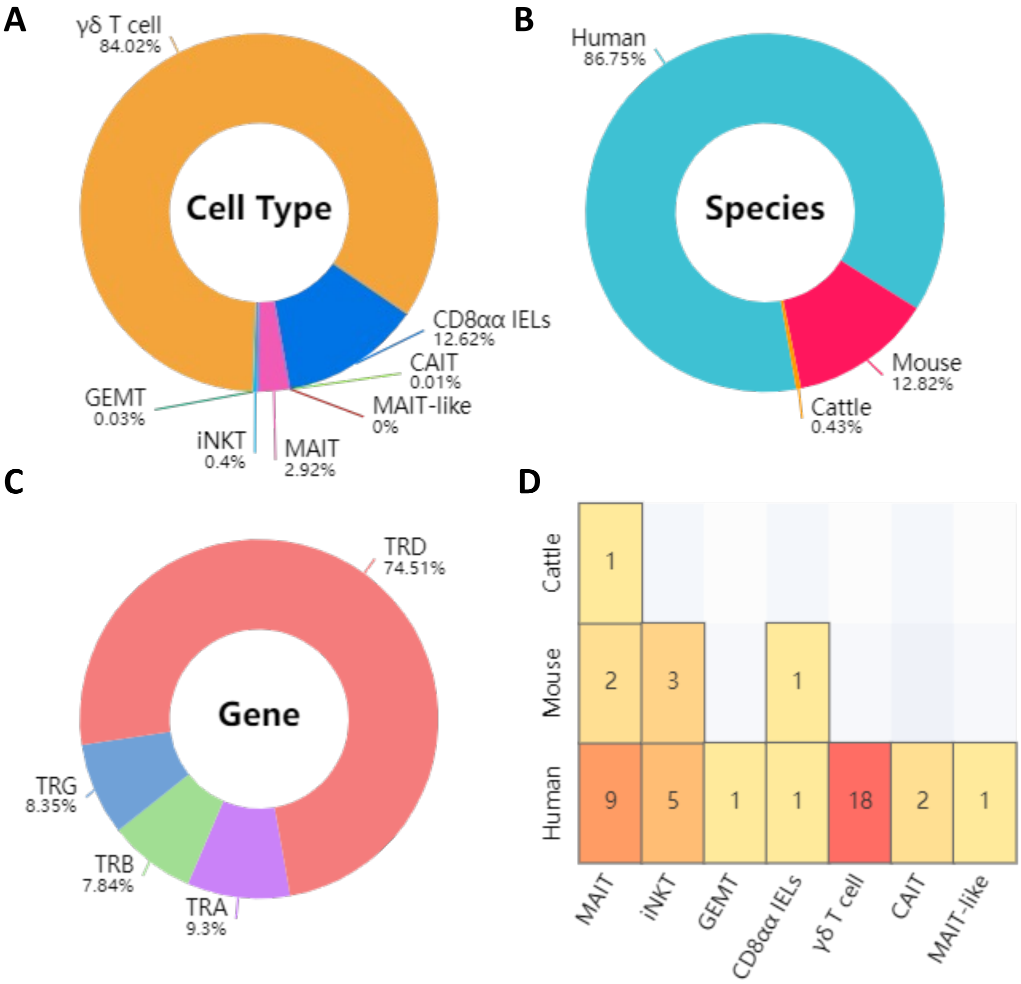

Supplementary Figure 1. UcTCRdb data statistics. Pie charts showing the summary of all TCR sequences by (A) cell subpopulation, (B) species, and (C) gene segments. (D) Heatmap of the number of different dataset sources in UcTCRdb.
